# Assessing Bone and Adipose Tissue Biomarkers in 5–6-Year-Old Polish Children Adhering to Vegetarian and Traditional Diets

**DOI:** 10.3390/nu18111653

**Published:** 2026-05-22

**Authors:** Jadwiga Ambroszkiewicz, Joanna Gajewska, Joanna Mazur, Grażyna Rowicka, Witold Klemarczyk, Magdalena Chełchowska

**Affiliations:** 1 Department of Screening Tests and Metabolic Diagnostics, Institute of Mother and Child, Kasprzaka 17a, 01-211 Warsaw, Poland; joanna.gajewska@imid.med.pl (J.G.); magdalena.chelchowska@imid.med.pl (M.C.); 2 Department of Humanization in Medicine and Sexology, Collegium Medicum, University of Zielona Gora, 65-729 Zielona Gora, Poland; j.mazur@cm.uz.zgora.pl; 3 Pediatric Gastroenterology Outpatient Clinic, Institute of Mother and Child, Kasprzaka 17a, 01-211 Warsaw, Poland; grazyna.rowicka@imid.med.pl; 4 Department of Nutrition, Institute of Mother and Child, Kasprzaka 17a, 01-211 Warsaw, Poland; witold.klemarczyk@imid.med.pl

**Keywords:** bone mineral density, bone metabolism markers, adipokines, vegetarian diet, preschool children

## Abstract

Background/Objectives: Plant-based diets are increasingly adopted by families with young children, yet their potential effects on bone development and metabolic regulation during early childhood remain insufficiently understood. This study aimed to evaluate body composition, bone mineral density (BMD), biochemical markers of bone turnover, and adipokine profiles in healthy children aged 5–6 years adhering to lacto-ovo-vegetarian or omnivorous diets. Methods: A cross-sectional analysis was conducted in a well-characterized cohort of 90 healthy normal-weight children consuming either lacto-ovo-vegetarian or omnivorous diets. Body composition and bone mineral density were measured using dual-energy X-ray absorptiometry, and circulating markers of bone formation, resorption, and adipokines were determined using ELISA methods. Correlation analyses were performed to examine the relationships between anthropometric variables, bone parameters, and adipokines. Results: No significant differences were observed between vegetarian and omnivorous diets in anthropometric characteristics, bone mineral content (BMC), or BMD, indicating comparable skeletal status. However, vegetarian children exhibited significantly higher levels of bone turnover markers, including bone alkaline phosphatase (BALP) (*p* = 0.023) and C-terminal telopeptide of type I collagen (CTX-I) (*p* = 0.035), and a lower osteocalcin OC/CTX-I ratio (*p* = 0.027). These findings may suggest a subtle imbalance in bone remodeling dynamics in these children, although their clinical significance remains uncertain. Additionally, higher levels of carboxylated osteocalcin (Gla-OC) (*p* = 0.022) and an increased carboxylated to undercarboxylated OC (Gla-OC/Glu-OC) ratio (*p* = 0.005) were observed in vegetarian children. Among adipokines, vegetarian children showed lower HMW adiponectin levels (*p* = 0.05) and a lower HMW/total adiponectin ratio (*p* = 0.012). Correlation analyses revealed distinct metabolic patterns between groups. In vegetarian children, bone parameters were primarily associated with lean mass, indicating the predominant role of mechanical factors in skeletal development. In contrast, omnivorous children demonstrated a more integrated relationship between bone indices and adipokines. Conclusions: In conclusion, while a lacto-ovo-vegetarian balanced diet supports normal bone mass in early childhood, it may be associated with subtle alterations in bone metabolism and its regulatory pathways, including adipokine profiles. These findings highlight the importance of adequate dietary planning and underscore the need for longitudinal studies to determine long-term effects on bone status.

## 1. Introduction

In recent years, interest in plant-based nutrition has grown rapidly among both adult and pediatric populations. Vegetarian diets, which exclude meat and, in some cases, other animal-derived products, are increasingly being adopted by families with young children for ethical, environmental, or perceived health-related reasons [1]. Although numerous studies in adults have demonstrated potential health benefits of vegetarian dietary patterns, including reduced risks of obesity, cardiovascular disease, hypertension, and type 2 diabetes mellitus [2,3,4,5], the implications of such dietary patterns during periods of rapid growth and development remain a subject of ongoing scientific debate [2,3,4,5].

Early childhood represents a critical period of intensive somatic growth, skeletal maturation, immune development, and metabolic programming [6]. Adequate nutrient intake during this period is essential to support optimal growth and long-term health outcomes. Particular attention has been directed toward bone metabolism and adipose tissue development, both of which are highly sensitive to dietary composition and nutrient availability. Bone accrual in childhood is fundamental for achieving optimal peak bone mass later in life, whereas adipose tissue functions not only as an energy store but also as an active endocrine organ involved in metabolic homeostasis [7,8].

Bone status is commonly assessed using bone mineral content (BMC) and bone mineral density (BMD), which reflect skeletal mineralization and bone strength. The measurement of biochemical markers of bone metabolism, including osteocalcin (OC) and its carboxylated (Gla-OC) and undercarboxylated (Glu-OC) forms, bone-specific alkaline phosphatase (BALP), and C-terminal telopeptide of type I collagen (CTX-I), provides valuable insights into the dynamic processes of bone formation and resorption [9,10].

The balance between bone formation and resorption is tightly regulated by complex molecular signaling pathways that control osteoblast and osteoclast differentiation and activity. Among the most important are the receptor activator of nuclear factor κB/receptor activator of nuclear factor κB ligand/osteoprotegerin (RANK/RANKL/OPG) system and the Wnt–β-catenin signaling pathway, which is modulated by sclerostin [11,12]. These pathways play central roles in skeletal remodeling and maintenance of bone mass.

In parallel, increasing attention has been paid to the role of adipose tissue metabolism and its endocrine function in early life [8,13]. Adipose tissue secretes a range of bioactive molecules, known as adipokines, including leptin and adiponectin, which regulate appetite, insulin sensitivity, inflammation, and energy metabolism. Importantly, adipokines also influence bone metabolism, highlighting the existence of a functional interplay between adipose tissue and the skeletal system [7]. A recent study has further demonstrated associations between genetic variability in leptin and bone mineral density in healthy young adults, thereby underscoring the biological relevance of leptin-related pathways in skeletal regulation [14]. Consequently, adipokines may represent valuable biomarkers of early alterations in body composition and metabolic health associated with dietary patterns.

A systematic review and meta-analysis conducted in adult populations suggested that vegetarian and, particularly, vegan diets may be associated with lower BMD at clinically relevant skeletal sites, including the femoral neck and lumbar spine, compared with omnivorous diets [15]. Similarly, large prospective cohort studies have reported an increased risk of both total and site-specific fractures among vegetarians and vegans relative to meat-eaters [16,17,18]. However, recent evidence derived from studies in young adults suggests a more heterogeneous relationship between vegetarian dietary patterns and skeletal health. Several studies have demonstrated comparable or even favorable bone outcomes among adolescents and young adults adhering to a vegetarian diet compared with omnivorous controls [19,20,21]. Vegetarian diets may also affect body composition and hydration-related parameters. A recent study in young adult women has shown associations between vegetarian diets and lower phase angle (PhA) values, as well as higher extracellular water to total body water (ECW/TBW) ratios assessed by bioelectrical impedance analysis, potentially reflecting subtle alterations in cellular integrity and body composition [22]. Collectively, these findings suggest that the skeletal effects of vegetarian diets may depend on multiple factors, including dietary quality, nutrient adequacy, developmental stage, and population-specific characteristics.

Despite the growing prevalence of vegetarian diets among children, relatively few studies have investigated their effects on skeletal development and metabolic regulation during early childhood. Existing pediatric evidence suggests that children following vegetarian diets may exhibit alterations in bone turnover markers and, in some cases, slightly lower BMC or BMD compared with their omnivorous peers [23,24]. However, the currently available studies are often limited by heterogeneous age ranges, inclusion of adolescents or young adults, relatively small sample sizes, and the lack of simultaneous assessment of both skeletal and metabolic biomarkers [19]. Moreover, data from European populations remain scarce, despite evidence indicating that the relationships between dietary patterns, bone metabolism, and body composition may be influenced by ethnicity, dietary habits, genetic background, and lifestyle-related factors [5,25,26,27].

Current recommendations regarding vegetarian diets in childhood also remain somewhat inconsistent. Several pediatric and nutrition societies state that well-planned vegetarian diets may be appropriate for children when carefully managed and adequately supplemented, whereas other organisations have raised concerns regarding the potential risk of inadequate nutrient intake and possible long-term consequences for skeletal health [28,29,30,31,32].

Given these considerations, there is a clear need for comprehensive studies focusing on narrow and developmentally critical age groups. The present analysis therefore focuses on a narrow and well-defined cohort of children aged 5–6 years, corresponding to the preschool period, which is characterized by relatively homogeneous developmental status and the occurrence of key developmental milestones.

This study aimed to address these important gaps by evaluating anthropometric parameters, body composition, bone mineral density, bone metabolism markers, and circulating adipokines in healthy children adhering to either a vegetarian or traditional omnivorous diet. Additionally, the study explored the potential associations between anthropometric measures and biochemical markers, offering a more integrated understanding of bone and adipose tissue metabolism in young children following vegetarian or omnivorous diets.

## 2. Materials and Methods

### 2.1. Participants

The study included 90 healthy children of Caucasian ethnicity aged 5–6 years. Participants’ health status was evaluated through medical history and basic physical examination. All studied children were of normal weight and free from chronic diseases that could affect bone metabolism. They followed balanced diets that met their energy and macronutrient requirements, in accordance with the Polish dietary guidelines [33]. Among them, 50 children (26 boys and 24 girls) had followed a vegetarian diet since birth, specifically a lacto-ovo-vegetarian dietary pattern that included milk, dairy products, and eggs. The parents independently sought specialized medical and nutritional care to evaluate their children’s nutritional status and to assess the adequacy of their growth and development while following a dietary pattern differing from standard Polish nutritional recommendations.

Approximately 75% of participants lived in urban areas with populations exceeding 100,000 inhabitants, whereas 6% resided in rural areas. A high level of maternal education was reported in 70% of cases, while 62% of fathers had attained higher education; secondary education was reported in 28% of both mothers and fathers. Overall, the participating families were characterized by a favorable socioeconomic status.

As previously described [34], prepubertal children following a vegetarian diet had a lower protein intake, higher carbohydrate intake, and comparable fat intake relative to omnivorous children. In the present study, both groups had similar total daily energy intake (1401 ± 368 vs. 1514 ± 432 kcal/day) and percentage of energy derived from fat (30.5 ± 5.3 vs. 31.6 ± 4.4%, *p* = 0.774). However, children on a vegetarian diet had a lower percentage of energy from protein (12.5 ± 2.4 vs. 15.7 ± 2.9%, *p* < 0.001) and a higher percentage of energy from carbohydrates (56.6 ± 5.4 vs. 52.0 ± 5.1%, *p* = 0.013) compared with omnivores.

The comparison group consisted of 40 children (20 boys and 20 girls) consuming a traditional omnivorous diet that included meat, poultry, and fish.

The exclusion criteria were low birth weight, developmental or nutritional disorders, gastrointestinal diseases, and regular medication use, with the exception of routine vitamin D supplementation at a dose of 600–1000 IU/day (15–25 μg/day), in accordance with updated Polish guidelines for the prevention and treatment of vitamin D deficiency [35].

Participants were recruited between July 2022 and June 2025 at the Department of Nutrition and Pediatric Gastroenterology Outpatient Clinic at the Institute of Mother and Child in Warsaw, Poland. According to parental interviews, all children demonstrated broadly comparable levels of habitual physical activity and were generally compliant with the 2020 World Health Organization (WHO) guidelines on physical activity and sedentary behavior for children and adolescents aged 5–17 years, which recommend at least 60 min of moderate-to-vigorous physical activity daily, including regular bone- and muscle-strengthening activities [36].

The study was conducted in accordance with the Declaration of Helsinki and approved by the Ethics Committee of the Institute of Mother and Child in Warsaw, Poland (Protocol Code: 15/2022; Date of Approval: 5 May 2022). Written informed consent was obtained from the parents or legal guardians of all participants.

### 2.2. Anthropometric and Body Composition Measurements

All participants underwent comprehensive clinical evaluation and anthropometric assessment. Height and weight were measured using standardized equipment, and body mass index (BMI) was calculated as body weight (kg) divided by height squared (m^2^). BMI Z-scores were derived using age- and sex-specific Polish reference charts [37]. Body composition, total body less head bone mineral content (TBLH-BMC), and bone mineral density in the total body less head (TBLH-BMD) and BMD in the lumbar spine (L1–L4) were assessed using dual-energy X-ray absorptiometry (DXA) with a Lunar Prodigy scanner (General Electric Healthcare, Madison, WI, USA) with pediatric database enCORE software version 9.30.044. We used TBLH-BMD according to the Official Positions of the International Society for Clinical Densitometry 2013 [38].

### 2.3. Biochemical Analyses

Fasting venous blood samples were collected between 8:00 a.m. and 10:00 a.m., centrifuged at 1000× *g* for 10 min at 4 °C to separate the serum, and stored at –80 °C until analysis. Serum levels of 25-hydroxyvitamin D (25(OH)D) were determined using the electrochemiluminescence method (ECLIA) with kits from DiaSorin Inc. (Stillwater, OK, USA). Biochemical bone metabolism markers and adipokine concentrations were quantified using commercially available human enzyme-linked immunosorbent assay (ELISA) kits according to the manufacturers’ protocols.

Bone metabolism markers:

- Bone alkaline phosphatase activity was measured using the BAP EIA kit (Quidel, Athens, OH, USA), with a detection limit of 0.7 U/L, and intra- and inter-assay coefficients of variation (CVs) below 5.8% and 7.6%, respectively.
- Osteocalcin and C-terminal telopeptide of type I collagen were assessed using N-MID Osteocalcin ELISA kits and the Serum CrossLaps (CTX-I) ELISA kit (IDS, Bolton, UK). The limit of detection for OC was 0.5 ng/mL, and intra- and inter-assay CVs were <2.2% and <5.1%, and for CTX-I were 0.02 ng/mL, <3.0% and <10.9%, respectively.
- Carboxylated and undercarboxylated osteocalcin levels were measured using ELISA kits from Takara Bio Inc. (Shiga, Japan), with intra- and inter-assay CVs below 2.4% and 4.8% for Gla-OC and <6.7% and 9.9% for Glu-OC, respectively. The limit of detection was 0.25 ng/mL for both forms of osteocalcin.
- Osteoprotegerin was determined using kits from DRG Diagnostics (Marburg, Germany), with a limit of detection of 0.03 pmol/L, intra-assay CV < 4.9% and inter-assay CV < 9.0%.
- Soluble receptor activator of nuclear factor kappa-B ligand was measured using the Human sRANKL ELISA kit from SunRed Biotechnology (Shanghai, China). The detection limit was 1.56 pg/mL; intra- and inter-assay CVs were <9% and <11%, respectively.
- Sclerostin was measured using the Sclerostin HS ELISA kit from Teco Medical Group (Sissach, Switzerland), with intra- and inter-assay CVs less than 4.8% and 8.2%, respectively, and a detection limit of 0.006 ng/mL.
- Adipokines:
- Leptin levels were determined using ELISA kits from DRG Diagnostics (Marburg, Germany), with a detection limit of 0.7 ng/mL and intra- and inter-assay CVs below 5.9% and 8.6%, respectively.
- Total adiponectin and high-molecular-weight adiponectin were measured using ELISA kits from ALPCO Diagnostics (Salem, NH, USA). The limit of quantitation was 0.019 ng/mL; intra- and inter-assay CVs were <5.4% and <5.0% for total adiponectin, and <5.0% and <5.7% for HMW adiponectin, respectively.

Biochemical parameters were measured in all children, except for HMW adiponectin, which was analyzed in 49 (98%) vegetarian and 38 (95%) omnivorous subjects.

### 2.4. Statistical Analyses

Normality of the data distribution was assessed using the Kolmogorov–Smirnov test. Data are presented as mean ± standard deviation (SD) for normally distributed variables and as median with interquartile range (IQR; 25th–75th percentiles) for non-normally distributed variables. For selected parameters, the relative percentage difference between the vegetarian and omnivore groups was calculated by dividing the absolute difference by the value in the second group and multiplying by 100. The ratios of OC/CTX-I, Gla-OC/Glu-OC, OPG/sRANKL, leptin/total adiponectin, and HMW/total adiponectin were calculated.

Group differences in anthropometric, biochemical, and bone-related parameters were evaluated using the Mann–Whitney U test. Whenever a statistically significant difference between groups was identified, the effect size (ES) was calculated as the absolute value of the standardized Mann–Whitney U test statistic divided by the square root of the total sample size (N = 90). According to commonly accepted guidelines, an effect size of up to 0.10 was considered small, up to 0.30 medium, and 0.50 or greater large. Correlation analyses were performed using Spearman’s rank correlation test. In addition, multivariate generalized linear models were constructed for selected parameters, including lean mass and five bone metabolism markers as independent variables. Estimates of the main effects are presented, and model fit was evaluated using the chi-square likelihood ratio omnibus test. This test determines whether inclusion of the predictors significantly improves model fit compared with the null model. Detailed model estimates are provided in Appendix A.

As is common in studies of this type, the sample size is determined by patient availability, which, in this case, are 5–6-year-old children who have followed a vegetarian diet since birth. Therefore, statistical power could only be evaluated post hoc. Power analysis was conducted using the free software G*Power (version 3.1.9.7), taking into account the type I error rate, group sizes, and effect size. With a total sample size of N = 90 and a type I error rate of α = 0.05, the achieved statistical power was 0.896 for a correlation effect size of r = 0.300 and 0.950 for a correlation of r = 0.337.

A *p*-value < 0.05 was considered statistically significant. All statistical analyses were conducted using IBM SPSS Statistics for Windows, version 29.0 (IBM Corp., Armonk, NY, USA).

## 3. Results

All participants were healthy, normal-weight Caucasian children aged approximately 5–6 years, who followed either a balanced lacto-ovo-vegetarian or an omnivorous diet. The groups were comparable with respect to age (vegetarians: 5.5 ± 0.5 years; omnivores: 5.6 ± 0.6 years) and sex distribution (vegetarians: 47% girls/53% boys; omnivores: 50% girls/50% boys).

### 3.1. Anthropometric and Body Composition Characteristics

The anthropometric and body composition characteristics of the participants are summarized in Table 1. No statistically significant between-group differences were observed in the basic anthropometric indices, including body weight, height, BMI, or BMI Z-score. Similarly, body composition parameters (fat mass and lean mass) did not differ significantly between the groups. However, omnivorous children showed a non-significant trend toward a higher fat mass percentage compared with vegetarians (21.4% vs. 17.6%, respectively).

Bone mineral outcomes were broadly comparable between the groups. Nevertheless, vegetarian children demonstrated slightly lower values across several bone parameters, including TBLH-BMC (−2.5%), spine BMC (−5.5%), TBLH-BMD (−3.5%), and lumbar spine BMD (L1–L4) (−3.5%). These differences did not reach statistical significance.

### 3.2. Biochemical Markers of Bone Metabolism and Adipokines

Several significant differences were observed between the groups of vegetarians and omnivores with respect to bone turnover markers and adipokines (Table 2).

Markers of bone formation and carboxylation status of osteocalcin differed between the groups. Vegetarian children had significantly higher BALP activity than omnivores. Total OC concentrations were similar between the groups; however, Glu-OC tended to be lower in vegetarians (*p* = 0.070), while Gla-OC was significantly higher (*p* = 0.022). As a result, the Gla-OC/Glu-OC ratio was significantly elevated in vegetarians (*p* = 0.005).

In parallel, serum CTX-I concentrations were significantly higher in vegetarian children. Consequently, the OC/CTX-I ratio was significantly lower in vegetarians. No significant between-group differences were found for serum levels of 25(OH)D, sclerostin, OPG, sRANKL, or the OPG/sRANKL ratio.

Among adipokines, leptin and total adiponectin levels were slightly lower in vegetarians, although these differences were not statistically significant. In contrast, vegetarian children displayed lower HMW adiponectin concentrations (*p* = 0.050) and a lower HMW/adiponectin ratio (*p* = 0.012).

**Figure 1:**
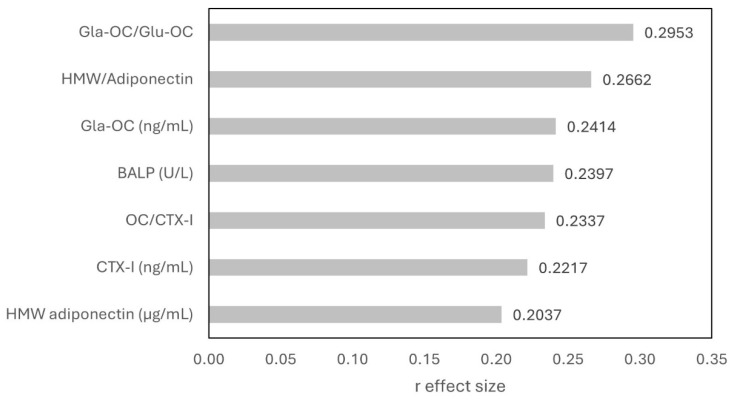
Mann–Whitney r effect size comparison of the two studied groups of children regarding biochemical parameters. BALP—bone alkaline phosphatase; OC—osteocalcin; Gla-OC—carboxylated osteocalcin; Glu-OC—undercarboxylated osteocalcin; CTX-I—C-terminal telopeptide of type I collagen; HMW-adiponectin—high molecular weight adiponectin.

The effect size (ES) was calculated for the seven parameters that differed significantly between the vegetarian and omnivore groups (Figure 1). Overall, the effects were small, with the largest ES observed for the Gla-OC/Glu-OC ratio (ES = 0.2953) and the HMW/adiponectin ratio (ES = 0.2662).

### 3.3. Correlation Analyses

The correlation analyses revealed distinct patterns of associations between the anthropometric variables, bone densitometry, bone turnover markers, and adipokines in vegetarian and omnivorous children (Table 3).

In both groups, anthropometric variables, particularly body weight and height, as well as lean mass, were strongly and consistently positively associated with bone outcomes, including total body and spine BMC and BMD. Fat mass also correlated with TBLH-BMC and TBLH-BMD, although these associations were generally weaker than those observed for lean mass.

Notable differences were observed in the relationships between biochemical markers and bone densitometric parameters. In vegetarian children, markers of bone turnover were positively related to bone density measures. Specifically, BALP and CTX-I levels correlated positively with spine BMC (*p* = 0.027 and *p* = 0.009, respectively). In addition, total OC (*p* = 0.018), Glu-OC (*p* = 0.034), and sclerostin (*p* = 0.027) concentrations were positively associated with lumbar spine BMD (L1–L4). In contrast, among omnivorous children, bone turnover markers were largely unrelated to densitometric indices, with the exception of OPG, which showed consistently negative correlations with BMC and BMD measures (*p* = 0.020 and *p* = 0.015, respectively).

Adipokines demonstrated the most pronounced differences between the studied groups. In vegetarians, adipokine concentrations were not significantly associated with bone parameters. Conversely, in omnivorous children, leptin showed strong positive associations (*p* < 0.001) with multiple bone mineral measures, including both total body and spine outcomes. Total adiponectin was strongly positively correlated with lumbar spine BMD (L1–L4) (*p* < 0.001), while HMW adiponectin was positively associated with TBLH-BMD (*p* = 0.025) and lumbar spine BMD (L1–L4) (*p* = 0.023).

**Figure 2:**
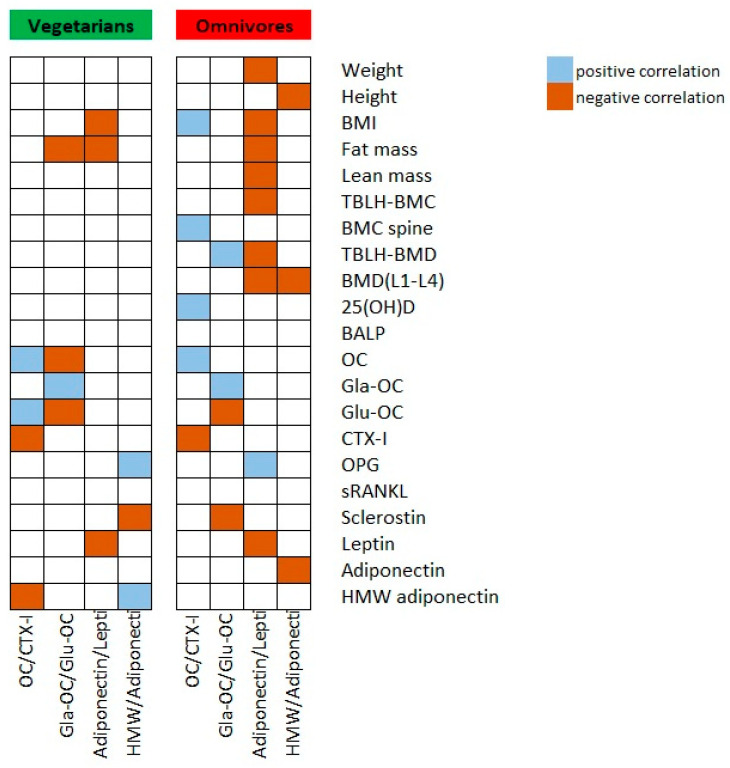
Partial Spearman correlations of bone metabolism markers and adipokine ratios with anthropometric and biochemical parameters in children following vegetarian and omnivorous diets. Data from 50 vegetarians and 40 omnivores were analyzed, and only statistically significant correlations with *p* ≤ 0.05 are shown. BMI—body mass index; TBLH—total body less head; BMC—bone mineral content; BMD—bone mineral density; BMD (L1–L4)—lumbar spine (L1–L4) bone mineral density; 25(OH)D—25-hydroxyvitamin D; OC—osteocalcin; Gla-OC—carboxylated osteocalcin; Glu-OC—undercarboxylated osteocalcin; CTX-I—C-terminal telopeptide of collagen type I; OPG—osteoprotegerin; sRANKL—soluble receptor activator of nuclear factor kappa-B ligand; HMW adiponectin—high molecular weight adiponectin.

Analyses of the ratio parameters provided additional insights into metabolic interactions (Figure 2).

In vegetarians, the OC/CTX-I ratio, which reflects bone formation to bone resorption processes, showed strong expected correlations with OC and CTX-I, but also a weak negative correlation with HMW adiponectin (r = −0.289, *p* = 0.042). The Gla-OC/Glu-OC ratio demonstrated expected correlations with OC fractions and was negatively correlated with fat mass (r = −0.329, *p* = 0.019). Adipokine ratios further reflected body composition links: the adiponectin/leptin ratio was negatively correlated with BMI (r = −0.280, *p* = 0.049) and fat mass (r = −0.374, *p* = 0.007). The HMW/adiponectin ratio correlated positively with OPG (r = 0.445, *p* = 0.001) and negatively with sclerostin (r = −0.348, *p* = 0.013) concentrations.

In omnivores, the ratio parameters showed broader and more integrated correlation patterns. The OC/CTX-I ratio correlated positively with BMI (r = 0.312, *p* = 0.050), TBLH-BMC (r = 0.353, *p* = 0.026), and 25(OH)D status (r = 0.372, *p* = 0.018). The Gla-OC/Glu-OC ratio was positively correlated with TBLH-BMD (r = 0.317, *p* = 0.047) and negatively with sclerostin concentration (r = −0.371, *p* = 0.019). The adiponectin/leptin ratio exhibited strong negative correlations with weight (r = −0.426, *p* = 0.006), BMI (r = −0.635, *p* < 0.001), fat mass (r = −0.311, *p* = 0.050), lean mass (r = −0.460, *p* = 0.003), TBLH-BMC (r = −0.548, *p* < 0.001), BMC spine (r = −0.512, *p* = 0.001), TBLH-BMD (r = −0.429, *p* = 0.006), and BMD (L1–L4) (r = −0.510, *p* = 0.001). This ratio was also negatively associated with OPG concentration (r = −0.323, *p* = 0.042). Finally, the HMW/adiponectin ratio was negatively associated with height (r = −0.400, *p* = 0.011) and BMD (L1–L4) (r = −0.417, *p* = 0.008).

### 3.4. Multivariate Analyses

Table 4 summarizes the main results of the generalized linear models estimated for five bone parameters in the vegetarian group. All models demonstrated satisfactory goodness-of-fit.

Lean mass emerged as the strongest predictor across the analyzed models. A significant association between Glu-OC and both TBLH-BMC and BMD (L1–L4) was observed. In the model estimated for spine BMC, the association with Glu-OC reached borderline statistical significance (*p* = 0.061). After adjustment for the remaining variables included in the analyses, CTX-I was significantly associated with spine BMC. Borderline associations for CTX-I were also found in the models for TBLH-BMC (*p* = 0.074) and BMD (L1–L4) (*p* = 0.070). In addition, sclerostin concentration was identified as a significant predictor of BMD (L1–L4) among children following a vegetarian diet. The detailed estimation results for all four models are provided in Table A1, Table A2, Table A3 and Table A4 in the Appendix A.

## 4. Discussion

The present study provides a comprehensive evaluation of bone mineral status, biochemical markers of bone turnover, and adipokine profiles in healthy children aged 5–6 years adhering to lacto-ovo-vegetarian and omnivorous diets. The main findings indicate that, despite comparable anthropometric characteristics and bone mineral density between the groups, vegetarian children exhibited subtle yet biologically relevant differences in bone remodeling markers and adipokine profiles. These observations suggest that dietary patterns may influence regulatory mechanisms of bone metabolism during early childhood, even when bone mass remains within the normal physiological range.

Consistent with previous pediatric research, body size and composition were the primary determinants of skeletal development [39,40]. Lean mass showed the strongest positive association with both bone mineral content and bone mineral density, confirming the central role of muscle-derived mechanical loading in bone modeling during growth. Furthermore, in our multivariate analyses, lean mass was identified as the strongest predictor across the analyzed models for both bone mineral content and bone mineral density. In agreement with earlier studies, vegetarian children (aged 5–10 years) have been reported to demonstrate comparable or only slightly lower spine BMC and BMD values, without clinically significant bone deficits [41,42]. Similarly, in the present study, bone mineral outcomes were generally comparable between the groups. Nevertheless, vegetarian children demonstrated modestly lower values (approximately 3.5–5.5%) across several skeletal parameters, including TBLH-BMC, spine BMC, TBLH-BMD, and lumbar spine BMD. Although these differences did not reach statistical significance, the consistent direction of the observed trends may be clinically relevant, as even small impairments in bone mineral accrual during childhood could potentially influence the attainment of optimal peak bone mass and long-term skeletal health. These findings warrant further investigation in larger prospective and longitudinal studies to determine whether such trends become more pronounced over time or during periods of accelerated skeletal growth. This interpretation is further supported by recent evidence indicating that vegetarian dietary patterns are not necessarily associated with impaired bone characteristics in young adults, suggesting that the skeletal effects of vegetarian diets may depend substantially on overall dietary quality, duration of dietary adherence, and lifestyle-related factors [Falbova].

The studies by Meyer and Protudjer [43] and Reis et al. [44] support the notion that a well-planned vegetarian diet, adequately supplemented, particularly with vitamin B_12_, and supervised by qualified healthcare professionals does not impair bone health during childhood. Nevertheless, the degree of dietary restriction and the child’s age remain critical determinants of nutritional risk [30,45].

An important observation of our study was the difference in the relationships between body composition and bone outcomes across the dietary groups. In vegetarian children, bone parameters were primarily associated with lean mass and body weight, whereas fat mass showed weaker and less consistent associations. This pattern suggests that bone development in this group may be driven predominantly by mechanical factors rather than adipose tissue-derived signaling pathways. In contrast, omnivorous children exhibited a more integrated pattern, in which both lean and fat mass contributed to bone parameters, indicating a broader and potentially more complex metabolic influence on skeletal regulation.

Despite comparable bone mineral status, vegetarian children in our study exhibited higher concentrations of bone turnover markers, including BALP and CTX-I, together with a lower OC/CTX-I ratio. This may suggest an imbalance in bone remodeling processes that is not yet reflected in current bone mass measurements. However, if sustained over time, it may have implications for long-term skeletal health. Similar findings have been reported in pediatric populations consuming plant-based diets, where elevated bone resorption markers and higher PTH concentrations suggest subtle alterations in bone metabolism emerging early in life [24,46]. The authors described a progressive increase in PTH concentrations across dietary groups (omnivorous < vegetarian < vegan), indicating a potential shift toward increased bone resorption with more restrictive plant-based diets. Importantly, these findings were not explained by differences in vitamin D status or calcium intake, both of which were adequate due to supplementation.

A recent study conducted by Itkonen et al. [24] included 2–7-year-old Finnish children and their caregivers on plant-based diets. Among children, no statistically significant differences were observed in primary bone turnover markers: tartrate-resistant acid phosphatase 5b (TRAP5b) and BALP, suggesting similar overall bone remodeling activity across dietary groups. Notably, these alterations appear to become more pronounced with age, as demonstrated in adult populations from similar cohorts. Vegetarian adults had elevated levels of either bone formation or bone resorption markers (BALP and TRAP5b), indicating an increase in bone turnover. Together with our findings of higher BALP and CTX-I levels, these observations suggest that subtle alterations in bone metabolism may already be present in early childhood and could potentially become more pronounced later in life. At the same time, vegetarian children in our study exhibited higher Gla-OC levels and a higher Gla-OC/Glu-OC ratio. Moreover, multivariate analyses demonstrated significant associations between Glu-OC and both TBLH-BMC and BMD (L1–L4). Overall, the observed effect sizes were relatively small; however, the most pronounced differences were identified for the Gla-OC/Glu-OC ratio. This may reflect subtle alterations in osteocalcin carboxylation status, as vitamin K is essential for the γ-carboxylation of osteocalcin, enabling its binding to hydroxyapatite and supporting bone mineralization [47].

However, both vitamin K intake and circulating vitamin K concentrations were not directly measured in the present study. Therefore, although some differences between groups were observed, their biological and clinical significance should be interpreted with caution, particularly in the context of the relatively limited sample size and the cross-sectional design of the study.

A novel aspect of this study is the analysis of adipokines in relation to bone parameters. Adipokines, such as leptin and adiponectin, are increasingly recognized as important mediators of the crosstalk between energy metabolism and skeletal homeostasis [7,8]. Experimental and clinical studies indicate that leptin can influence bone metabolism both centrally through hypothalamic pathways and peripherally through direct effects on osteoblasts and osteoclasts [48,49]. Recent evidence further supports the involvement of leptin-related pathways in mineral metabolism, as genetic variation within leptin rs7799039 has been associated with total body mineral estimates independently of fat mass parameters in young healthy adults [14].

This is consistent with studies conducted in pediatric populations, including prepubertal children and adolescents, where leptin has been shown to correlate positively with bone mass, particularly in individuals with normal or higher adiposity [50,51,52,53]. Although leptin concentrations did not differ significantly between our studied groups, its association with bone parameters varied. In omnivorous children, leptin was positively associated with BMD, particularly at the lumbar spine and total body, consistent with its proposed anabolic role. In contrast, no such associations were observed in our group of vegetarian children, suggesting that the role of leptin in bone metabolism in these children may be modulated by other factors such as body composition, energy availability, and the metabolic context.

Differences were also observed in adiponectin profiles, with vegetarian children exhibiting lower levels of HMW adiponectin, which is the most biologically active isoform. Although the role of adiponectin in bone metabolism remains complex and not fully understood, several studies have reported inverse associations between adiponectin levels and BMD and its influence on bone remodeling [13,48,54,55]. The lower HMW adiponectin level and HMW/adiponectin ratio observed in our group of vegetarian children may indicate subtle differences in adipose-bone signaling; however, the clinical significance of this finding remains unclear and warrants further investigation.

Our analysis of ratio-based biomarkers provides additional insights into the integrated regulation of both bone and metabolic pathways. In vegetarian children, these ratios showed more selective associations, mainly with bone turnover markers, supporting a more compartmentalized regulatory pattern. Additionally, the associations between the HMW/total adiponectin ratio and regulatory markers such as OPG and sclerostin may indicate a potentially protective but indirect role of adipokine isoforms in bone regulation in children on a vegetarian diet. In contrast, omnivorous children exhibited broader associations, linking these ratios not only with bone turnover but also with anthropometric parameters and vitamin D status. The adiponectin-related ratios were consistently negatively associated with both anthropometric and bone parameters. This suggests a more integrated interaction between the skeletal and metabolic pathways in children consuming mixed diets.

Overall, our findings highlight the differences in the regulation of bone metabolism rather than bone mass status in 5–6-year-old vegetarian and omnivorous children. A more mechanically driven and selectively regulated pattern of bone development, accompanied by increased remodeling activity, was observed in children on a vegetarian diet. In contrast, a more integrated metabolic profile involving adipokines, bone turnover markers, and body composition was observed in children following a traditional diet.

The main strength of this study is the simultaneous comprehensive assessment of DXA-derived bone parameters, body composition, biochemical bone metabolism markers, and adipokines in a well-characterized cohort of 5–6-year-old children. This integrative approach allowed for the evaluation of both structural and metabolic aspects of bone health and provided insights into bone–adipose tissue interactions. The inclusion of children adhering to a lacto-ovo-vegetarian diet from early life represents a unique and valuable contribution, reported here for the first time. Additionally, the study groups were well matched for age, sex, and anthropometric parameters, minimizing potential confounding factors.

Several limitations of this study should be acknowledged. First, its cross-sectional design precludes any inference of causality. Second, the relatively small sample size and single-center setting limit the statistical power and generalizability of the findings, as well as restricting the ability to examine more complex interactions between variables. Nevertheless, our study focuses on a narrow and well-defined cohort of children aged 5–6 years, corresponding to the preschool period, which is characterized by relatively homogeneous developmental status; this reduces potential confounding related to heterogeneity in growth and maturation. In addition, the vegetarian subgroup adhered to a clearly defined lacto-ovo-vegetarian dietary pattern, which improved internal comparability and minimized heterogeneity associated with different types of vegetarian diets. All anthropometric and biochemical measurements were obtained at a single time point. Third, detailed dietary intake data, including micronutrients, vitamins, and amino acid profiles, were not available. Only basic macronutrient distribution (percentage of energy from protein, fat, and carbohydrates) was assessed, limiting the precision of nutrient–biomarker relationships. Fourth, physical activity was not assessed in detail using standardized questionnaires or objective monitoring tools. Although parental reports indicated broadly comparable habitual physical activity levels between groups and general adherence to WHO recommendations, residual confounding due to differences in physical activity cannot be excluded. Finally, despite these limitations, the study’s main strength lies in the well-characterized and homogeneous cohort, which is rarely achievable in research involving healthy prepubertal children. Accordingly, while the findings should be interpreted with caution, they may provide a useful basis for future longitudinal studies investigating the relationships between diet, bone metabolism, and metabolic health in early childhood.

## 5. Conclusions

In conclusion, children aged 5–6 years following vegetarian or omnivorous diets exhibit comparable growth, body composition, and bone mineral density, indicating that well-planned vegetarian diets do not adversely affect skeletal development in early childhood. However, observed differences in bone turnover markers and adipokine–bone interactions suggest distinct regulatory patterns of bone metabolism between dietary groups. Vegetarian children demonstrate an imbalance in bone turnover and a more selective, mechanically driven pattern of skeletal regulation, whereas omnivorous children display a more metabolically integrated profile involving adiposity-related signals. These findings highlight the importance of combining densitometric, bone metabolism markers and adipokine assessments when evaluating bone health in children and underscore the need for careful nutritional planning in plant-based diets during early life. Future longitudinal studies are warranted to further clarify the long-term implications of these dietary patterns for skeletal development and metabolic health.

## Figures and Tables

**Table 1:** Anthropometric and body composition parameters of children on vegetarian and omnivorous diets.

	Vegetarians (n = 50)	Omnivores (n = 40)	*p*
Weight (kg)	19.1 ± 2.1	19.7 ± 3.4	0.791
Height (cm)	114.9 ± 4.5	115.3 ± 5.6	0.569
BMI (kg/m^2^)	14.4 ± 1.1	14.7 ± 1.6	0.448
BMI Z-score	−0.529 ± 0.888	−0.345 ± 1.064	0.436
Fat mass (%)	17.6 (15.0–21.5)	21.4 (16.5–24.2)	0.133
Fat mass (kg)	3.18 (2.67–3.76)	3.35 (2.79–4.45)	0.294
Lean mass (kg)	14.30 ± 1.89	14.98 ± 2.86	0.318
TBLH-BMC (g)	607 ± 115	621 ± 154	0.789
BMC spine (g)	49.8 ± 10.9	52.7 ± 11.8	0.338
TBLH-BMD (g/cm^2^)	0.586 ± 0.050	0.607 ± 0.055	0.078
TBLH-BMD Z-score	−0.382 ± 0.852	−0.318 ± 0.676	0.758
BMD (L1–L4) (g/cm^2^)	0.573 ± 0.068	0.593 ± 0.052	0.259
BMD (L1–L4) Z-score	−0.740 ± 0.942	−0.605 ± 0.593	0.302

Data are presented as mean value ± SD or median and IQR; BMI—body mass index; TBLH—total body less head; BMC—bone mineral content; BMD—bone mineral density; BMD (L1–L4)—lumbar spine (L1–L4) bone mineral density.

**Table 2:** Serum concentrations of bone metabolism markers and adipokines in children on vegetarian and omnivorous diets.

	Vegetarians	Omnivores	*p*
Bone metabolism markers			
25(OH)D (ng/mL)	27.3 ± 9.7	29.1 ± 6.8	0.408
BALP (U/L)	130.5 (93.4–160.6)	112.6 (90.6–128.3)	0.023
OC (ng/mL)	73.6 (56.4–94.2)	70.2 (62.7–90.7)	0.955
Gla-OC (ng/mL)	33.9 (28.6–38.6)	30.2 (23.0–35.9)	0.022
Glu-OC (ng/mL)	24.7 (15.5–35.9)	30.1 (24.6–38.1)	0.070
CTX-I (ng/mL)	1.947 ± 0.495	1.695 ± 0.580	0.035
OPG (pmol/L)	4.57 ± 0.92	4.62 ± 0.93	0.890
sRANKL (ng/mL)	2036 (692–3726)	1729 (1176–3111)	0.782
Sclerostin (ng/mL)	0.424 ± 0.123	0.436 ± 0.096	0.470
OC/CTX-I	37.6 (27.4–51.6)	46.2 (37.9–55.9)	0.027
Gla-OC/Glu-OC	1.37 (0.87–2.45)	1.01 (0.74–1.32)	0.005
OPG/sRANKL	0.002 (0.001–0.004)	0.003 (0.001–0.005)	0.881
Adipokines			
Leptin (ng/mL)	1.40 (0.82–1.85)	1.54 (0.77–3.15)	0.342
Adiponectin (µg/mL)	9.16 ± 2.26	9.66 ± 2.87	0.567
HMW adiponectin (µg/mL)	5.79 ± 1.75	6.48 ± 1.90	0.050
Adiponectin/leptin	7.14 (4.08–10.18)	6.71 (3.07–10.38)	0.470
HMW/Adiponectin	63.0 ± 8.9	67.8 ± 8.8	0.012

Data are presented as mean ± SD or median and IQR; 25(OH)D—25-hydroxyvitamin D; BALP—bone alkaline phosphatase; OC—osteocalcin; Gla-OC—carboxylated osteocalcin; Glu-OC—undercarboxylated osteocalcin; CTX-I—C-terminal telopeptide of type I collagen; OPG—osteoprotegerin; sRANKL—soluble receptor activator of nuclear factor kappa-B ligand; HMW-adiponectin—high molecular weight adiponectin.

**Table 3:** Bivariate associations between densitometry parameters (BMC, BMD) and anthropometry and biochemical markers (bone markers, adipokines) in children on vegetarian and omnivorous diets.

	Vegetarians				Omnivores			
	TBLH-BMC	BMC Spine	TBLH-BMD	BMD (L1–L4)	TBLH-BMC	BMC Spine	TBLH BMD	BMD (L1–L4)
Weight	0.730 0.000	0.775 0.000	0.549 0.000	0.584 0.000	0.703 0.000	0.742 0.000	0.504 0.001	0.579 0.000
Height	0.676 0.000	0.691 0.000	0.602 0.000	0.490 0.000	0.561 0.000	0.646 0.000	0.384 0.014	0.452 0.004
BMI	0.344 0.014	0.436 0.002	0.463 0.001	0.314 0.026	0.673 0.000	0.569 0.000	0.489 0.001	0.422 0.007
Fat mass	0.163 0.257	0.368 0.009	0.342 0.015	0.419 0.002	0.373 0.019	0.387 0.015	0.339 0.035	0.366 0.022
Lean mass	0.757 0.000	0.606 0.000	0.710 0.000	0.334 0.018	0.652 0.000	0.611 0.000	0.621 0.000	0.500 0.001
25(OH)D	0.092 0.525	0.054 0.616	0.052 0.625	0.155 0.284	−0.119 0.463	−0.090 0.583	0.083 0.611	−0.096 0.583
BALP	0.252 0.078	0.312 0.027	0.255 0.074	0.164 0.256	0.186 0.252	0.012 0.940	0.048 0.768	0.018 0.914
OC	0.086 0.553	0.116 0.421	0.097 0.501	0.333 0.018	0.026 0.873	0.126 0.438	0.054 0.739	0.259 0.111
Gla-OC	0.172 0.234	0.176 0.221	0.165 0.252	0.130 0.369	0.145 0.371	0.013 0.936	0.070 0.668	0.076 0.644
Glu-OC	0.007 0.960	0.040 0.785	0.011 0.941	0.300 0.034	−0.144 0.375	0.008 0.962	−0.218 0.176	−0.061 0.710
CTX-I	0.198 0.169	0.367 0.009	0.320 0.024	0.234 0.102	−0.169 0.296	−0.240 0.136	−0.215 0.183	−0.041 0.805
OPG	0.118 0.415	0.109 0.451	0.174 0.227	0.135 0.350	−0.367 0.020	−0.374 0.017	−0.381 0.015	−0.215 0.183
sRANKL	0.044 0.762	0.159 0.271	0.109 0.450	0.235 0.350	−0.232 0.150	−0.280 0.081	−0.038 0.814	−0.023 0.888
Sclerostin	0.147 0.308	0.106 0.463	0.128 0.374	0.313 0.027	0.065 0.692	0.030 0.854	−0.079 0.628	0.151 0.359
Leptin	−0.007 0.962	−0.033 0.822	−0.094 0.517	−0.054 0.712	0.618 0.000	0.564 0.000	0.535 0.000	0.326 0.043
Total adiponectin	−0.029 0.841	0.148 0.304	0.053 0.716	−0.003 0.981	0.181 0.265	0.279 0.081	0.238 0.139	0.532 0.000
HMW adiponectin	0.020 0.890	0.151 0.294	0.075 0.603	−0.067 0.644	0.099 0.542	0.240 0.135	0.353 0.025	0.362 0.023

Data are presented as Spearman’s rho and *p* values; TBLH—total body less head; BMC—bone mineral content; BMD—bone mineral density; BMI—body mass index; 25(OH)D—25-hydroxyvitamin D; BALP—bone alkaline phosphatase; OC—osteocalcin; Gla-OC—carboxylated osteocalcin; Glu-OC—undercarboxylated osteocalcin; CTX-I—C-terminal telopeptide of type I collagen; OPG—osteoprotegerin; sRANKL—soluble receptor activator of nuclear factor kappa-B ligand; HMW-adiponectin—high molecular weight adiponectin.

**Table 4:** Main effects obtained in the multivariate generalized linear models estimated in the vegetarian group (significance level: *p* < 0.05).

	Dependent Variable			
	TBLH-BMC	BMC Spine	TBLH-BMD	BMD (L1–L4)
Independent variable				
(Constant)	0.0035	0.0267	<0.0001	0.0025
BALP (U/L)	0.1955	0.4006	0.9962	0.6687
OC (ng/mL)	0.1553	0.5518	0.6493	0.3282
Glu-OC (ng/mL)	0.0061	0.0617	0.3491	0.0108
CTX-I	0.0742	0.0031	0.0626	0.0707
Sclerostin (ng/mL)	0.3217	0.6901	0.7091	0.0020
Lean mass (kg)	<0.0001	<0.0001	<0.0001	0.0020
Likelihood ratio omnibus test				
chi-square	59.245	37.165	47.075	21.698
df	6	6	6	6
*p*	<0.0001	<0.0001	<0.0001	0.0014

df—degrees of freedom; TBLH—total body less head; BMC—bone mineral content; BMD—bone mineral density; BALP—bone alkaline phosphatase; OC—osteocalcin; Glu-OC—undercarboxylated osteocalcin; CTX-I—C-terminal telopeptide of type I collagen.

## Data Availability

All data generated and analyzed in this study are included in this article. Further inquiries are available upon request from the corresponding author.

## Appendix A

**Table A1:** Generalized linear model for TBLH-BMC in the vegetarian group (N = 50).

Independent Variable	B	Standard Error	95% Wald Confidence Interval		Wald Chi-Square	df	*p*
			Lower Bound	Upper Bound			
(Constant)	−251.107	85.9728	−419.611	−82.604	8.531	1	0.003
BALP (U/L)	0.306	0.2364	−0.157	0.769	1.676	1	0.195
OC (ng/mL)	−0.907	0.6381	−2.158	0.344	2.020	1	0.155
Glu-OC (ng/mL)	2.706	0.9869	0.771	4.640	7.516	1	0.006
CTX-I	36.486	20.4339	−3.563	76.536	3.188	1	0.074
Sclerostin (ng/mL)	77.797	78.4987	−76.058	231.651	0.982	1	0.322
Lean mass (kg)	0.049	0.0051	0.039	0.060	93.050	1	<0.001

**Table A2:** Generalized linear model for spine BMC in the vegetarian group (N = 50).

Independent Variable	B	Standard Error	95% Wald Confidence Interval		Wald Chi-Square	df	*p*
			Lower Bound	Upper Bound			
(Constant)	−22.457	10.1373	−42.326	−2.589	4.908	1	0.027
BALP (U/L)	0.023	0.0279	−0.031	0.078	0.707	1	0.401
OC (ng/mL)	−0.045	0.0752	−0.192	0.103	0.354	1	0.552
Glu-OC (ng/mL)	0.217	0.1164	−0.011	0.446	3.491	1	0.062
CTX-I	7.123	2.4094	2.400	11.845	8.739	1	0.003
Sclerostin (ng/mL)	3.690	9.2560	−14.451	21.832	0.159	1	0.690
Lean mass (kg)	0.004	0.0006	0.002	0.005	34.911	1	0.000

**Table A3:** Generalized linear model for TBLH-BMD in the vegetarian group (N = 50).

Independent Variable	B	Standard Error	95% Wald Confidence Interval		Wald Chi-Square	df	*p*
			Lower Bound	Upper Bound			
(Constant)	0.247	0.0424	0.164	0.330	33.832	1	<0.001
BALP (U/L)	5.49 × 10^−7^	0.0001	0.000	0.000	0.000	1	0.996
OC (ng/mL)	0.000	0.0003	−0.001	0.000	0.207	1	0.649
Glu-OC (ng/mL)	0.000	0.0005	0.000	0.001	0.877	1	0.349
CTX-I	0.019	0.0101	−0.001	0.039	3.468	1	0.063
Sclerostin (ng/mL)	0.014	0.0388	−0.061	0.090	0.139	1	0.709
Lean mass (kg)	2.06 × 10^−5^	2.53 × 10^−6^	1.56 × 10^−5^	2.55 × 10^−5^	66.059	1	<0.001

**Table A4:** Generalized linear model for BMD (L1–L4) in the vegetarian group (N = 50).

Independent Variable	B	Standard Error	95% Wald Confidence Interval		Wald Chi-Square	df	*p*
			Lower Bound	Upper Bound			
(Constant)	0.223	0.0738	0.078	0.367	9.121	1	0.003
BALP (U/L)	8.68 × 10^−5^	0.0002	0.000	0.000	0.183	1	0.669
OC (ng/mL)	−0.001	0.0005	−0.002	0.001	0.956	1	0.328
Glu-OC (ng/mL)	0.002	0.0008	0.000	0.004	6.497	1	0.011
CTX-I	0.032	0.0175	−0.003	0.066	3.266	1	0.071
Sclerostin (ng/mL)	0.148	0.0674	0.016	0.280	4.818	1	0.028
Lean mass (kg)	1.36 × 10^−5^	4.403 × 10^−6^	4.97 × 10^−6^	2.22 × 10^−5^	9.539	1	0.002
